# OXA-830, a Novel Chromosomally Encoded Extended-Spectrum Class D β-Lactamase in *Aeromonas simiae*

**DOI:** 10.3389/fmicb.2019.02732

**Published:** 2019-11-26

**Authors:** Qianqian Chen, Wangxiao Zhou, Changrui Qian, Kai Shen, Xinyi Zhu, Danying Zhou, Zhewei Sun, Wei Lu, Hongmao Liu, Kewei Li, Teng Xu, Qiyu Bao, Junwan Lu

**Affiliations:** ^1^School of Laboratory Medicine and Life Science/Institute of Biomedical Informatics, Wenzhou Medical University, Wenzhou, China; ^2^Tongji University School of Medicine, Shanghai, China; ^3^Institute of Translational Medicine, Baotou Central Hospital, Baotou, China; ^4^School of Medical and Health, Lishui University, Lishui, China

**Keywords:** oxacillinase, OXA-830, *Aeromonas simiae*, resistance, kinetic analysis

## Abstract

The diversity of class D β-lactamases mediating resistance to β-lactams has been increasingly reported recently. In this study, a novel class D oxacillinase named OXA-830 was identified in a fully sequenced *Aeromonas simiae* strain, which was isolated from sewage discharged from a farm in southern China. OXA-830 shares the highest amino acid identity of 79.3% with an OXA-12-like variant named OXA-725. When expressed in *E. coli* DH5α, OXA-830 conferred resistance to penicillins and selected β-lactamase inhibitors but not to cephalosporins and carbapenems. Kinetic analysis of OXA-830 revealed a broad substrate profile including penicillins, cefazolin, cefoxitin, and ceftazidime but not carbapenems. The hydrolytic activity was significantly inhibited by sulbactam, followed by tazobactam, but was less effectively inhibited by clavulanic acid. The *bla*_OXA–__830_ gene was located on the *A. simiae* A6 chromosome and the *bla*_OXA–__830_-related region was bracketed by a pair of perfect inverted repeats.

## Introduction

The genus *Aeromonas* is a distinct group of oxidase-positive, facultatively anaerobic, Gram-negative bacilli of the family *Aeromonadaceae* (Colwell et al., 1986). Members of *Aeromonas* can be isolated from every environmental niche where bacterial ecosystems exist, including aquatic habitats and fish as well as food products, and are implicated in human and animal infections (Janda and Abbott, 2010). The species *Aeromonas simiae* was first described in two strains (CIP 107798 and CIP 107797) isolated from feces of healthy monkeys from Mauritius (Harf-Monteil et al., 2004). Further study revealed that these two strains may originate from the same clone because they share entirely identical 16S rRNA, *gyrB*, and *rpoD* genes with each other (Saavedra et al., 2006). To date, there have been a large number of reports about the presence of β-lactamase genes among *Aeromonas* strains (Carvalho et al., 2012; Chen et al., 2012).

Oxacillin-hydrolyzing (OXA)-type β-lactamases (OXAs) constitute most of the members of Ambler class D active-serine-site β-lactamases (Bush et al., 1995) and are widely identified among clinically relevant Gram-negative bacteria, such as *Pseudomonas* spp., *Acinetobacter* spp., *Aeromonas* spp. and *Enterobacteriaceae* (Poirel et al., 2010). Most of the OXAs possess the ability to hydrolyze cloxacillin or oxacillin at a rate of >50% that for benzylpenicillin and are typically not inhibited by commercially available β-lactamase inhibitors such as clavulanic acid, tazobactam, and sulbactam (Payne et al., 1994; Bush et al., 1995). According to the Bush functional classification scheme for β-lactamases (Bush et al., 1995), OXAs are classified into group 2d. Although most of the early identified OXAs exhibited a substrate profile strictly restricted to penicillin, oxacillin, cloxacillin and nitrocefin, several OXA members have been demonstrated to be active against extended-spectrum cephalosporins which is typically due to a small number of point mutations occurring in parental narrow-spectrum class D β-lactamases (DBLs), such as the derivatives of OXA-10 (Poirel et al., 2010; Leonard et al., 2013). For instance, compared to OXA-10, OXA-11 (N146S and G167D) exhibits the ability to hydrolyze ceftazidime (Hall et al., 1993); OXA-17 (N76S) has an increased hydrolytic ability for cefotaxime as well as a decreased capacity for ceftazidime (Danel et al., 1999); and OXA-19, which differed from OXA-10 by nine amino acids, can hydrolyze ceftazidime with a low activity (Mugnier et al., 1998). Some OXAs have evolved to exhibit hydrolytic activity toward β-lactams of “last resort,” i.e., carbapenems (El Garch et al., 2011; Antonelli et al., 2015).

Similar to other antimicrobial resistance genes, many OXA β-lactamase genes have been identified on both plasmids and chromosomes with diverse mobile genetic elements (MGEs), such as integrons, insertion sequences and transposons (Poirel et al., 2010). For example, the *bla*_OXA–__1_-like, *bla*_OXA–__2_-like, and *bla*_OXA–__10_-like genes were commonly captured as gene cassettes by integrons in plasmids (Naas and Nordmann, 1999); the *bla*_OXA–__23_ gene from the chromosome of *Acinetobacter radioresistens* may be transferred onto plasmids diffusing into *Acinetobacter baumannii* through the IS*Aba1*-based composite transposon Tn*2006* or transposon-like structure named Tn*2008*, or a single copy of IS*Aba4* upstream of the gene (Corvec et al., 2007; Adams-Haduch et al., 2008); and the *bla*_OXA–__58_ gene-encoding regions in *A. baumannii* isolated from different countries (France, Spain, Romania, and Turkey) were bracketed by IS*Aba3* on one side and IS*Aba3*, IS*Aba1* or IS*18* on the other side (Poirel and Nordmann, 2006). However, according to the literature, there are intrinsic chromosomally encoded OXAs in many bacterial species (Poirel et al., 2010). The first such gene, identified in 1994, originated from the chromosome of *Aeromonas jandaei* (formerly *Aeromonas sobria*), which was not associated with an integron or transposon and was named *bla*_OXA–__12_ (Rasmussen et al., 1994).

In this study, for the first time, we determined the complete genome sequence of *A. simiae*, i.e., a sewage-derived *A. simiae* strain A6. Based on sequence analysis, we identified and characterized a novel chromosomally encoded DBL named OXA-830, which is quite divergent from the other OXA β-lactamases.

## Materials and Methods

### Bacterial Strains

The host strain *A. simiae* A6 carrying the *bla*_OXA–__830_ gene was isolated in November 2017 from sewage discharged from a farm in Wenzhou, China. Species identification was conducted using the Vitek-60 microorganism autoanalysis system (BioMérieux Corporate, Craponne, France). Further verification was performed based on the 16S rRNA sequencing method. Moreover, considering limited resolution the 16S rRNA gene provides to discriminate among closely related species of the genus *Aeromonas* (Alperi et al., 2008), a multilocus phylogenetic analysis (MLPA) of the concatenated sequences of 6 housekeeping genes (*gyrB*, *rpoD*, *recA*, *dnaJ*, *gyrA*, and *atpD*) as previously reported (Beaz-Hidalgo et al., 2015) as well as whole-genome sequence-based phylogenetic analysis using kSNP3.0 (Gardner et al., 2015) were conducted to determine the evolutionary relationship of *A. simiae* A6 with 32 other *Aeromonas* sp. strains of different species. Two neighbor-joining phylogenetic trees were generated by using MEGA7 with 1,000 bootstrap replications (Kumar et al., 2016). The bacteria and plasmids used in this work are listed in Supplementary Table S1.

### Antimicrobial Susceptibility Testing

The minimum inhibitory concentrations (MICs) were determined using the agar dilution method following the guidelines of the Clinical and Laboratory Standards Institute (CLSI). Susceptibility patterns were interpreted according to the CLSI breakpoint criteria (CLSI, 2019) and the guidelines of the European Committee on Antimicrobial Susceptibility Testing [EUCAST] (2019) for *Enterobacteriaceae*. No interpretation criteria were available for benzylpenicillin, benzylpenicillin/clavulanic acid, benzylpenicillin/sulbactam, oxacillin, cloxacillin and polymyxin B from CLSI (2019) and European Committee on Antimicrobial Susceptibility Testing [EUCAST] (2019). *E. coli* ATCC 25922 was used as a reference strain for quality control.

### Whole-Genome Sequencing and Sequence Analysis

Whole-cell DNA of *A.simiae* A6 was extracted using the AxyPrep Bacterial Genomic DNA Miniprep kit (Axygen Scientific, Union City, CA, United States) and sequenced on a PacBio RS II instrument (Pacific Biosciences). The PacBio long reads were initially assembled by Canu v1.6 (Koren et al., 2017), and then two FASTQ sequence files generated using the Illumina HiSeq 2500 platform were mapped onto the primary assembly to control assembly quality and to correct possible misidentified bases by using Bwa and the Genome Analysis Toolkit (McKenna et al., 2010). The consensus sequence was obtained by a custom-derived script written in Python^1^. Potential open reading frames (ORFs) were predicted using Glimmer software (Delcher et al., 2007) and annotated against the UniProt/Swiss-Prot and non-redundant protein databases using the BLASTX program with an *e*-value threshold of 1e-5. GView was used to construct basic genomic features (Petkau et al., 2010). Annotation of MGEs and resistance genes was performed using ISfinder (Siguier et al., 2006), INTEGRALL (Moura et al., 2009) and ResFinder (Zankari et al., 2012) with default parameters. The molecular weight and pI value of OXA-830 was predicted using ProtParam^2^. The putative signal peptide cleavage site of OXA-830 was identified by SignalP 5.0 (Almagro Armenteros et al., 2019). Amino acid alignment and the neighbor-joining phylogenetic tree construction of OXA-830 with other DBLs were performed using the MAFFT program and MEGA7 with a bootstrap of 1,000 replicates, respectively (Katoh and Standley, 2013; Kumar et al., 2016). Comparisons of the nucleotide sequences were performed using BLASTN. Other bioinformatics tools were written using Python (see text footnote 1) and Biopython (Cock et al., 2009).

### Cloning of the *bla*_OXA–__830_ Gene and Expression and Purification of OXA-830

The gene encoding OXA-830 along with its promoter was amplified by PCR using forward (5′-CGGAATTCAGACACAGATTGGCACAGCA-3′) and reverse (5′-CCCAAGCTTGCCCGGTGAAGAAGAAGTGA-3′) primers with a pair of flanking restriction endonuclease adapters. Then, the PCR product was eluted from an agarose gel, digested with the *Eco*RI and *Hin*dIII, and ligated into the pUCP24 vector with a T4 DNA ligase cloning kit (Takara Bio, Inc., Dalian, China). The recombinant plasmids were transformed into competent *E. coli* DH5α by the calcium chloride method, and bacterial colonies were cultured on Luria-Bertani agar plates supplemented with 20 μg/mL gentamicin. The recombinant plasmids were verified by both restriction enzyme digestion and Sanger sequencing (Shanghai Sunny Biotechnology Co., Ltd., Shanghai, China). The similar procedure was also applied to cloned complete ORF of *bla*_OXA–__830_ into pET-28b. The recombinant plasmid (pET-OXA-830) was transformed into competent *E. coli* BL21 cells by the calcium chloride method, and transformants were selected on Luria-Bertani agar plates supplemented with 50 μg/mL kanamycin. The authenticity of cloned fragments was confirmed by Sanger sequencing (Shanghai Sunny Biotechnology Co., Ltd., Shanghai, China). The above-mentioned *E. coli* BL21 transformant was grown in Luria-Bertani medium with 50 μg/mL kanamycin at 37°C. Overnight cultures were diluted 100-fold in 200 mL of Luria-Bertani medium and incubated for hours at 37°C with orbital shaking. Isopropyl-β-d-thiogalactopyranoside (IPTG) (Sigma Chemicals Co., St. Louis, MO, United States) was added to a final concentration of 1 mM until the cultures reached an OD_600_ between 0.6 and 0.8, and incubation was continued for an additional 4 h. OXA-830 was isolated from the periplasm and purified by affinity chromatography based on the instructions of the His-tag Protein Purification Kit (P2226, Beyotime, China).

### Determination of Kinetic Parameters

Kinetic parameters for hydrolysis of β-lactams by the purified OXA-830 β-lactamase were examined using a UV-VIS spectrophotometer (U-3900, HITACHI, Japan) at 30°C in 10 mM phosphate buffer (pH 7.0) in a final reaction volume of 300 μL. The steady-state kinetic parameters (*k*_cat_ and *K*_*M*_) were determined by non-linear regression of the initial reaction rates with the Michaelis–Menten equation in Prism (version 7) software (GraphPad Software, San Jose, CA, United States).

β-lactamase inhibition was studied with benzylpenicillin (500 μM) as the substrate. The β-lactamase inhibitors sulbactam, tazobactam and clavulanic acid at various concentrations were preincubated with the purified OXA-830 β-lactamase for 3 min at 30°C before addition of substrate. The inhibitor concentration required to reduce the hydrolysis of 500 μM benzylpenicillin by 50% was determined by non-linear regression with the log(inhibitor) vs. response – Variable slope equation in Prism (version 7) software (GraphPad Software, San Jose, CA, United States).

### Nucleotide Sequence Accession Number

The complete nucleotide sequences of the chromosome of *A. simiae* A6 and the *bla*_OXA–__830_ gene in this work have been submitted to DDBJ/EMBL/GenBank under accession numbers CP040449 and MK926981, respectively.

## Results and Discussion

### Identification and Characterization of the OXA-830-Producing Isolate, *A. simiae* A6

**FIGURE 1:**
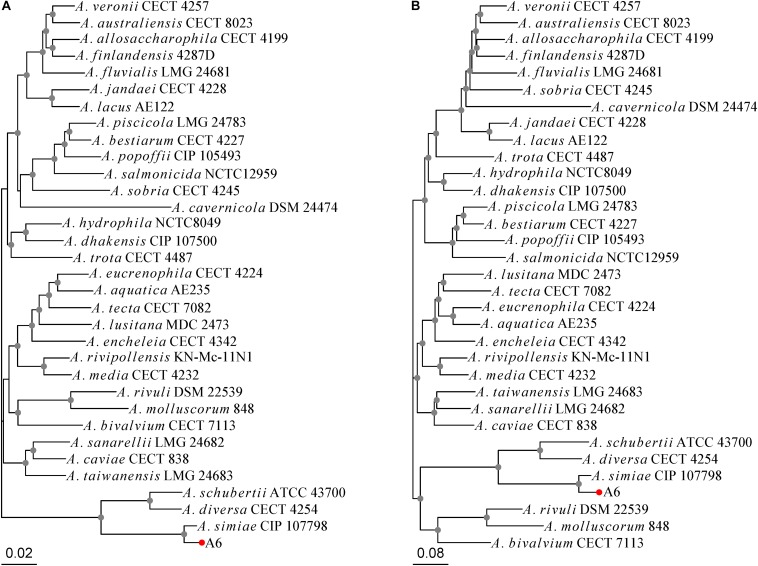
Phylogenetic relationships of *A. simiae* A6 with all other *Aeromonas* species inferred on the **(A)** MLPA tree and **(B)** whole-genome-based phylogeny tree. Nodes supported with bootstrap values over 50% are marked by gray dots. *A. simiae* A6 is indicated with a red dot.

*Aeromonas simiae* A6 was isolated in 2017 from sewage discharged from a farm in Wenzhou, southern China. A 16S ribosomal RNA gene homology analysis showed that *A. simiae* A6 had the closest relationship with one *A. simiae* strain (*A. simiae* IBS S6874 [NR_025585.1]), at 99% identity and 99% coverage. The MLPA tree and whole-genome-based phylogeny tree congruously showed that *A. simiae* A6 was phylogenetically closest to *A. simiae* CIP 107798, which constituted a robust phylogenetic branch with considerable reliability (Figures 1A,B). We finally grouped the strain into the species *A. simiae* and named it *A. simiae* A6.

*In vitro* susceptibility testing showed that wild-type *A. simiae* A6 exhibited resistance to ampicillin and ticarcillin (Table 1). Moreover, the MICs of oxacillin, cloxacillin, benzylpenicillin, benzylpenicillin/clavulanic acid and benzylpenicillin/sulbactam against *A. simiae* A6 were 512, 512, 64, 64, and 32 μg/mL, respectively, which were significantly higher than or equal to the resistance breakpoint for ampicillin (≥32 μg/mL), though no interpretation criteria for these antimicrobials were available.

**TABLE 1:** Minimum inhibitory concentrations of 32 antimicrobials for 5 strains (mg/L).

**Antibiotic**	**A6**	**pUCP24-*bla*_OXA–__830_/DH5α**	**pUCP24/DH5α**	**DH5α**	**ATCC 25922**
Benzylpenicillin	64	1,024	32	16	32
Benzylpenicillin-clavulanic acid	64	256	32	16	32
Benzylpenicillin-tazobactam	16	128	16	16	16
Benzylpenicillin-sulbactam	32	64	32	16	16
Ampicillin	128	512	4	4	4
Ampicillin-sulbactam	16	16	2	2	2
Oxacillin	512	>2,048	512	512	256
Cloxacillin	512	>2,048	512	512	256
Ticarcillin	64	512	1	2	4
Ticarcillin-clavulanic acid	64	256	2	4	2
Piperacillin	8	256	1	2	2
Piperacillin-tazobactam	1	4	1	2	2
Cefazolin	4	2	4	2	2
Cefoxitin	2	2	4	2	2
Ceftazidime	1	0.125	0.125	0.25	0.25
Cefepime	0.5	0.06	0.06	0.125	0.125
Aztreonam	1	0.125	0.06	0.06	0.25
Imipenem	0.125	0.25	0.5	0.25	0.25

**FIGURE 2:**
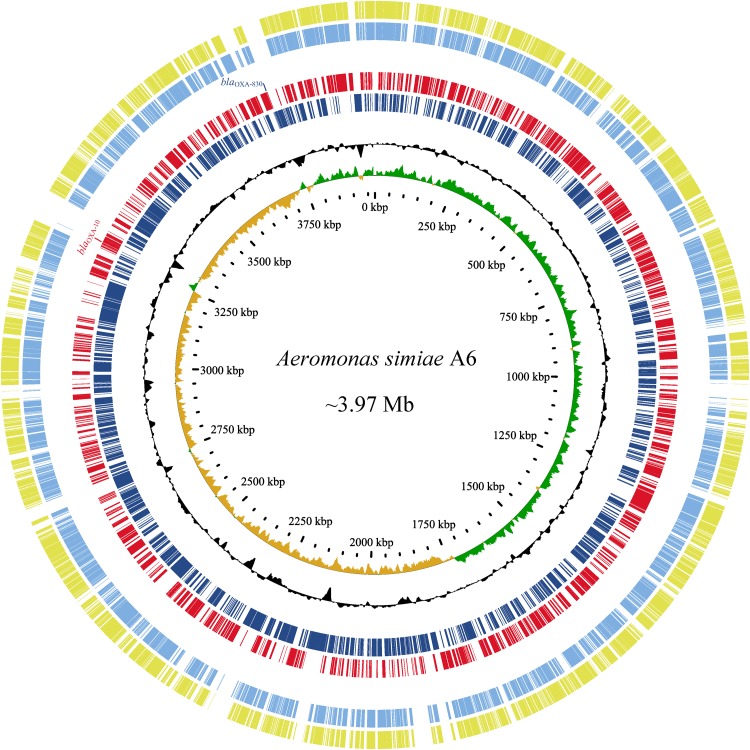
Genomic comparison of *A. simiae* A6 with its close relatives. From outside to inside: circles 1 and 2 are homologous regions of *A. schubertii* WL1483 (accession number CP013067) and *A. schubertii* LF1708 (CP039611) compared to *A. simiae* A6, while unmatched regions are left blank; circles 3 and 4 display predicted ORFs encoded in the forward strand and reverse strand, respectively; circles 5 and 6 represent the GC content and GC skew maps, respectively; and circle 7 shows the scale in kb.

Since the high MICs of the aforementioned antimicrobials and no complete genome sequence of species *A. simiae* is currently available in the public database, the complete genome of *A. simiae* A6 was determined to unveil the potential factors associated with the resistance profiles. The results showed that *A. simiae* A6 has a circular chromosome (without plasmids) of 3.97 Mb in size that contains 3,633 ORFs with an average GC content of 60.56% (Table 2 and Figure 2). Comparative genomics analysis revealed the genome of *A. simiae* A6 shared the highest sequence similarities with that of *A. schubertii* WL1483 (accession number CP013067, at 91.62% identity and 65% coverage) and *A. schubertii* LF1708 (accession number CP039611, at 91.56% identity and 65% coverage) (Figure 2). Notably, *A. simiae* A6 encodes two predicted DBL-encoding genes. One is *bla*_OXA–__10_ located in a truncated class I integron with a gene array of *intI1*-*qnrVC4*-*cmlA5*-*bla*_OXA–__10_-*aac(6’)-Ib-cr*-*aadA1*-*dfrA14*-*mobC*-IS*6100* (ranging from 3261 to 3269 kb), and the other belongs to a novel OXA β-lactamase named *bla*_OXA–__830_, sharing the top amino acid identity (79.3%) with a function-known β-lactamase gene *bla*_OXA–__12_ variant called *bla*_OXA–__725_ (X80276.1) in the database.

**TABLE 2:** General features of the *A. simiae* A6 genome.

	**Chromosome**
Size (bp)	3,974,097
GC content (%)	60.56
Predicted coding sequences (CDSs)	3,633
Known proteins	3,149 (86.7%)
Hypothetical proteins	484 (13.3%)
Protein coding (%)	87.41%
Average ORF length (bp)	956
Average protein length (aa)	317
rRNA operons	(16S-23S-5S)^∗^9
	16S-23S-5S-5S
tRNAs	126

### Possible Origin of the New Class D β-Lactamase OXA-830

**FIGURE 3:**
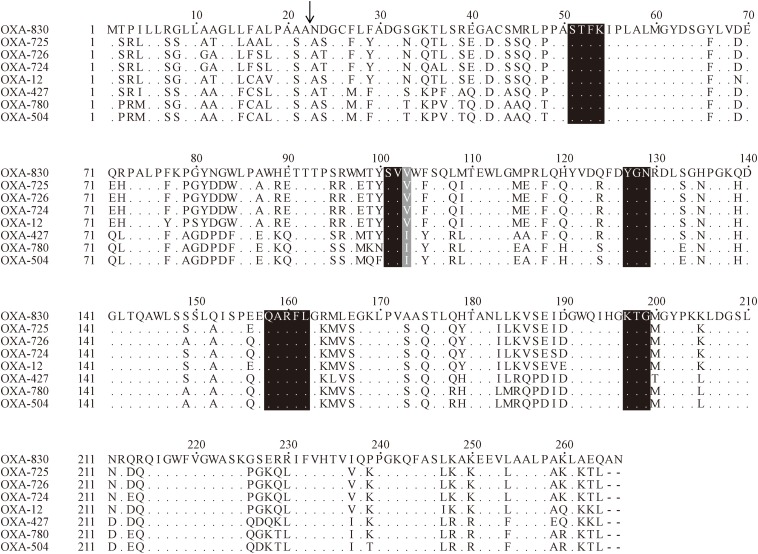
Amino acid alignment of OXA-830 with other selected class D β-lactamases. Dots indicate amino acid residues identical to those of OXA-830. Conserved motifs are shaded in black, while the poorly conserved site at residue 120 (DBL numbering) is shaded in gray. The predicted signal sequence cleavage site for OXA-830 is indicated by a vertical arrow.

*bla*_OXA–__830_ gene was 801 bp in length and encoded a 266 amino acid preprotein of ca. 29.4 kDa. A signal peptide cleavage site was predicted to be between an alanine and asparagine at amino acid residues 22 and 23. In addition, the pI value of OXA-830 was predicted to be 9.18. The closest relative of OXA-830 was OXA-725, a class D β-lactamase with detectable hydrolytic activity against oxacillin, penicillin and ampicillin as well as carbenicillin, which was previously described in a clinical *A. jandaei* (formerly *A. sobria*) isolate from the Hammersmith Hospital, London (Walsh et al., 1995). Screening for *bla*_OXA–__830_-homologous genes (>70% amino acid sequence identity) in the NCBI nucleotide database showed that most of the close relatives (89.19%, 66/74) were derived from the genus *Aeromonas*, except 4 genes from *Enterobacteriaceae* and 4 of undetermined origin, suggesting the importance of *Aeromonas* as the reservoir for *bla*_OXA–__830_-like genes. Additionally, OXA-830 was distantly related to other class D β-lactamases in amino acid sequence identity, i.e., OXA-12 and its close variants (77.1% with OXA-12; 78.9% with OXA-726; and 78.6% with OXA-724). Moreover, it shared identities of respective 72.2, 71.1, and 70.7% with OXA-427, OXA-780, and OXA-504 (Figure 3), and possessed less than 50% identities to all of the other DBLs.

**FIGURE 4:**
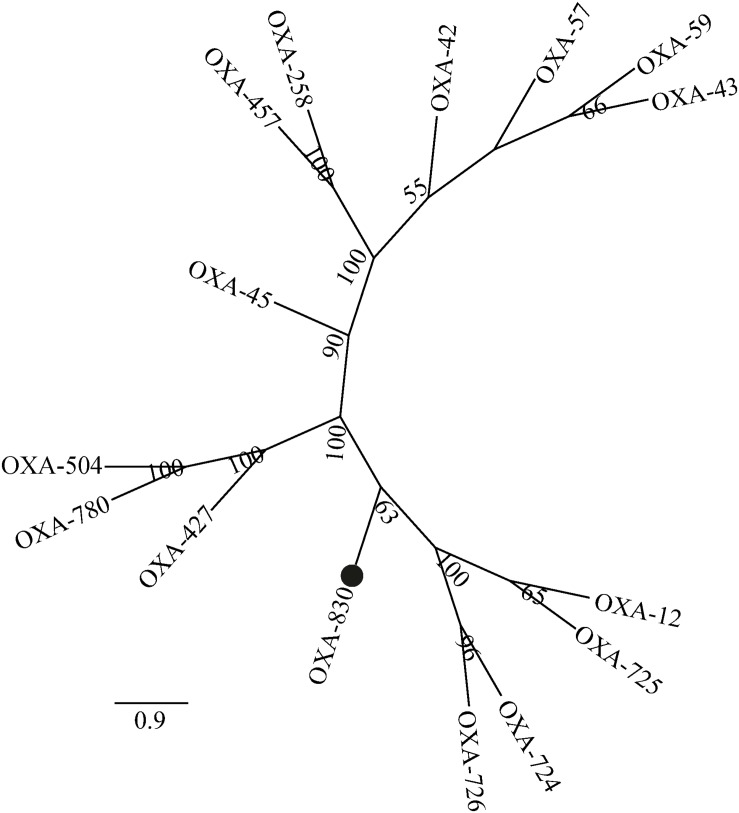
A phylogenetic tree showing the relationship of OXA-830 with other homologous DBLs. Bootstrap values are shown at branch points. OXA-830 from our study is indicated with a filled circle.

Four conserved motifs were identified from OXA-830 and other OXA family DBLs: serine-threonine-phenylalanine-lysine (S-T-F-K) at positions 70 to 73, tyrosine-glycine-asparagine (Y-G-N) at positions 144 to 146, Q-X-X-X-L at positions 176 to 180, and lysine-threonine-glycine (K-T-G) at positions 216 to 218 (DBL numbering). However, the conserved S-X-V motif at residues 118 to 120 (DBL numbering), one of the active sites of DBLs (Paetzel et al., 2000), was found to be slightly different in OXA-427, OXA-780, and OXA-504, whose valine at residue 120 was replaced by isoleucine. In addition, OXA-830 possessed as many as 40 unique amino acid differences compared to all the other DBLs (Figure 3). A phylogenetic tree containing OXA-830 and other DBLs (>41% amino acid identity) (Figure 4) was constructed and the results showed that OXA-830 clustered closest to OXA-12, OXA-725, OXA-726, and OXA724. Thus, OXA-830 represents a novel lineage of DBLs.

### Genetic Context of *bla*_OXA–__830_ Gene

**FIGURE 5:**
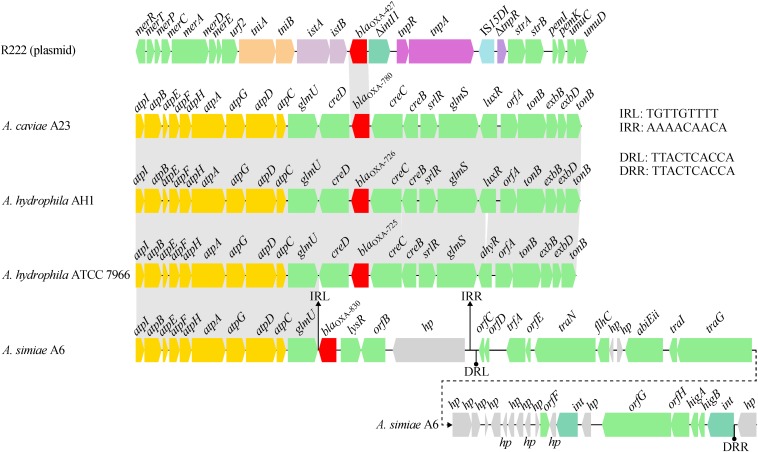
Comparison of the genetic context of the *bla*_OXA–830_ gene with that of selected β-lactamase genes. Genes are shown as arrows and colored based on gene function classification. Shading denotes regions of homology (>85% nucleotide sequence identity). Genes without direct gene names are illustrated as *orfA*, chromosome segregation ATPase; *orfB*, NADH:flavin oxidoreductase/NADH oxidase; *orfC*, AlpA family phage regulatory protein; *orfD* and *orfE*, DNA-binding proteins; *orfF*, Permease; *orfG*, DEAD/DEAH box helicase; and *orfH*, virulence associated protein. Regions are drawn to scale from accession numbers CP000462.1 (*A. hydrophila* ATCC 7966 chromosme), NZ_LSZC01000019.1 (*A. hydrophila* AH1 chromosome), LFXO01000139.1 (*A. caviae* A23 chromosome), and KX869741.1 (plasmid R222 from *E. cloacae* 20130723).

A comparative genomics analysis of the genetic environment of the *bla*_OXA–__830_ gene in A6 with that of closely related DBL-encoding genes in three other *Aeromonas* strains showed that *glmU* (encoding the bifunctional protein GlmU) together with upstream sequences shared a conserved syntenic architecture in gene content and gene order (Figure 5). However, the genes downstream of *glmU* in the *bla*_OXA–__830_-related region were highly distant to those of all the other *bla*_OXA_ genes. In fact, the region from *bla*_OXA–__830_ to a large hypothetical gene in the *bla*_OXA–__830_-related region was flanked by a pair of perfect 9-bp inverted repeats (IRs), suggesting these genes might be transferred from the other strain through horizontal gene transfer. Furthermore, an approximately 25-kb genomic island including two phage integrase-encoding genes was found upstream of the large hypothetical gene, which was enwrapped by a pair of perfect 10-bp direct repeats (DRs). This indicated that the mobilization of this genomic island onto the genome of A6 might have resulted from the integrase-mediated site-specific recombination. Altogether, these potential lateral gene transfer events may well explain the discrepancy of gene content between the *bla*_OXA–__830_-related region and other *bla*_OXA_-related regions.

### Functional Characterization of the OXA-830 β-Lactamase

The cloned *bla*_OXA–__830_ gene in *E. coli* DH5α recombinant clones (pUCP24-*bla*_OXA–__830_/DH5α) conferred resistance to all the penicillins and β-lactamase inhibitors examined in this study except for benzylpenicillin/sulbactam (Table 1). More specifically, it facilitated different increases in MIC levels for ampicillin (128-fold), ampicillin/sulbactam (8-fold), oxacillin (>4-fold), cloxacillin (>4-fold), benzylpenicillin (32-fold), benzylpenicillin/tazobactam (8-fold), benzylpenicillin/clavulanic acid (8-fold), ticarcillin (512-fold), ticarcillin/clavulanic acid (128-fold), piperacillin (256-fold) and piperacillin/tazobactam (4-fold) compared with those of the control strain (pUCP24/DH5α), although the MICs against ampicillin, benzylpenicillin, ticarcillin, and piperacillin were decreased in the presence of inhibitors. In addition, OXA-830 did not confer resistance to cephalosporins, aztreonam or carbapenems (Table 1).

The kinetic parameters of the OXA-830 β-lactamase purified from the extract of *E. coli* BL21 harboring the recombinant plasmid pET-OXA-830 demonstrated that OXA-830 had a strong hydrolytic activity against penicillins (including oxacillin, cloxacillin, benzylpenicillin, ampicillin, piperacillin, and ticarcillin) and cefazolin (*k*_cat_/*K*_*m*_ ratios were ≥0.73 μM^–1^⋅s^–1^), while very poor hydrolytic activities were detected for cefoxitin and ceftazidime. Moreover, no measurable hydrolytic activities were observed for cefepime, aztreonam and imipenem (Table 3). Nevertheless, this finding was not entirely consistent with the above MIC changes for *E. coli* DH5α recombinant clones (pUCP24-*bla*_OXA–__830_/DH5α) in the antibiotic susceptibility assay, as the hydrolytic activities against cefazolin, cefoxitin and ceftazidime did not result in significant changes in the MICs for *E. coli* DH5α recombinant clones. It may be that the activity *in vitro* was simply too low to contribute to activity *in vivo*. A similar phenomenon was observed from OXA-436 which showed hydrolytic activity against ceftazidime but not confer resistance to ceftazidime (Samuelsen et al., 2018). OXA-830 exhibited an approximately 2-fold increase in the turnover rate (*k*_cat_) for oxacillin and cloxacillin compared to that for benzylpenicillin. The highest catalytic efficiency was observed with ampicillin (*k*_cat_/*K*_*m*_ ratio was 2.36 μM^–1^⋅s^–1^). Of note, the catalytic efficiencies of OXA-830 against oxacillin and ampicillin matched or surpassed those of several clinically important DBLs in Gram-negative pathogens, such as OXA-48 and OXA-58 (Docquier et al., 2009; Verma et al., 2011). Compared to OXA-12 (Rasmussen et al., 1994), OXA-830 exhibited significantly lower affinity for penicillins used in both studies. Although susceptible to cephalosporins, the extended hydrolysis spectrum of OXA-830 indicated that it could be exceptionally classified into subgroup 2de, a new subgroup of DBLs including the oxacillin- or cloxacillin-hydrolyzing β-lactamases exhibiting hydrolytic activity against oxyimino-cephalosporins but not carbapenems (Bush and Jacoby, 2010). Interestingly, most of the members of this subgroup originated from OXA-10, with a small number of amino acid substitutions described as previously (Bush and Jacoby, 2010). However, OXA-830 exhibited only 22.6% global amino acid identity with OXA-10. In contrast, OXA-830 shared much higher global amino acid identity (72.2%) with OXA-427, which could hydrolyze imipenem and exhibit resistance to extended-spectrum cephalosporins (mostly ceftazidime) (Bogaerts et al., 2017).

**TABLE 3:** Kinetic parameters of various β-lactam antibiotics for the purified OXA-830 β-lactamase.

**Substrate**	***K*_*M*_ (μM)**	***k*_cat_ (s^–1^)**	***k*_cat_/*K*_*M*_ (μM^–1^⋅s^–1^)**
Benzylpenicillin	540	527	0.98
Ampicillin	188	444	2.36
Oxacillin	722	1239	1.72
Cloxacillin	737	1033	1.40
Ticarcillin	208	227	1.09
Piperacillin	233	413	1.77
Cefazolin	975	715	0.73
Cefoxitin	76	2.79	0.037
Ceftazidime	61	2.04	0.033
Cefepime	NH^∗^	NH	NH
Aztreonam	NH	NH	NH
Imipenem	NH	NH	NH

*Data are the means of three independent measurements. Standard deviations were always within 10% of the mean values. ^∗^NH, no detectable hydrolysis.*

The results of β-lactamase activity inhibition measurement, as measured by IC_50_s (50% inhibitory concentrations), showed that OXA-830 was strongly inhibited by sulbactam (IC_50_: 0.05 ± 0.01 μM) and tazobactam (IC_50_: 0.07 ± 0.02 μM), and was less sensitive to clavulanic acid (IC_50_: 0.26 ± 0.02 μM), which could explain the above MIC changes of β-lactams with or without β-lactamase inhibitors. However, this finding was inconsistent with what was found for OXA-12 (Rasmussen et al., 1994), which was strongly inhibited by clavulanic acid (IC_50_: 0.009 μM) and tazobactam (IC_50_: 0.03 μM), and less effectively inhibited by sulbactam (IC_50_: 0.24 μM).

## Conclusion

The complete genome sequence of *A. simiae* was firstly reported in this work. We identified a novel OXA DBL named OXA-830 that was encoded on the chromosome of *A. simiae* A6. The new enzyme exhibited high amino acid sequence divergence from all currently available DBLs and showed the top amino acid identities (77.1–79.3%) with OXA-12 and OXA-12-like β-lactamases. Therefore, OXA-830 could be characterized as the first member of a new lineage of DBLs. OXA-830 showed some common functional properties with other OXA β-lactamases such as OXA-12, but significant differences were also observed. It possessed extended-spectrum hydrolytic properties which was strongly inhibited by both sulbactam and tazobactam. These findings revealed the importance of OXA-830 as a new model to study the structure/function relationships among OXA β-lactamases. Paired terminal IRs at both ends of the *bla*_OXA–__830_-related region suggested that the possibility of dissemination of *bla*_OXA–__830_ in the environment is still existent, although it is not associated with any known MGE.

## Data Availability Statement

The datasets generated for this study can be found in the GenBank database as GenBank: CP040449 for the *A. simiae* A6 genome sequence, GenBank: MK926981 for the OXA-830 gene.

## Author Contributions

QC, KS, XZ, DZ, HL, WL, and KL collected the strains and performed the experiments. WZ, ZS, and QC analyzed the experimental results. WZ, QC, and TX performed the bioinformatics analysis. WZ, JL, and QB co-led the writing of the manuscript. JL and QB designed the work. All authors read and approved the final version of the manuscript.

## Conflict of Interest

The authors declare that the research was conducted in the absence of any commercial or financial relationships that could be construed as a potential conflict of interest.

## Footnotes

1 https://www.python.org/

2 https://web.expasy.org/protparam/
