# Supplementary material for: OXA-830, a Novel Chromosomally Encoded Extended-Spectrum Class D β-Lactamase in Aeromonas simiae
Source: Front Microbiol. 2019 Nov 26;10:2732. doi: 10.3389/fmicb.2019.02732 (PMC6902050; doi:10.3389/fmicb.2019.02732)
Supplement: Supplementary file 1 [file Data_Sheet_1.docx]

**Table S1. Bacteria and plasmids used in this work**

| Strain or plasmid | Relevant characteristic(s)* | Reference or source |
| --- | --- | --- |
| Strain |  |  |
| A6 | The wild-type strain of Aeromonas simiae A6 | This study |
| DH5α | *Escherichia coli* DH5α was used as a host for the cloning of the blaOXA-830 gene | Our laboratory collection |
| BL21 | *E. coli* BL21 was used as a host for expression and purification of OXA-830 | Our laboratory collection |
| ATCC 25922 | *E. coli* ATCC 25922 was used as a quality control for antimicrobial susceptibility testing | Our laboratory collection |
| pUCP24-blaOXA-830/DH5α | DH5α carrying the pUCP24 vector encoding the blaOXA-830 gene with its upstream promoter region | This study |
| pET28b-blaOXA-830/BL21 | BL21 carrying the pET28b vector encoding the *bla*_OXA-830_ gene | This study |
| Plasmid |  |  |
| pUCP24 | Cloning vector for the PCR products of the blaOXA-830 gene with its upstream promoter region; GENr | Our laboratory collection |
| pET28b | Expression vector for the PCR products of the blaOXA-830 gene | Our laboratory collection |

*GEN, gentamicin; r, resistance.
